# Evidence supporting regulatory-decision making on orphan medicinal products authorisation in Europe: methodological uncertainties

**DOI:** 10.1186/s13023-018-0926-z

**Published:** 2018-11-15

**Authors:** Caridad Pontes, Juan Manuel Fontanet, Roser Vives, Aranzazu Sancho, Mònica Gómez-Valent, José Ríos, Rosa Morros, Jorge Martinalbo, Martin Posch, Armin Koch, Kit Roes, Katrien Oude Rengerink, Josep Torrent-Farnell, Ferran Torres

**Affiliations:** 1 Departament de Farmacologia, de Terapèutica i de Toxicologia, Unitat Docent Parc Taulí, Universitat Autònoma de Barcelona, C/Parc Taulí, 1, 08208 Sabadell, Spain; 2 Departament de Farmacologia, de Terapèutica i de Toxicologia, Universitat Autònoma de Barcelona, Unitat Docent de Sant Pau, C/St Antoni Maria Claret 167, 08025 Barcelona, Spain; 3 Unitat de Farmacologia Clínica, Institut d’Investigació i Innovació Parc Taulí I3PT, Parc Taulí Hospital Universitari, Universitat Autònoma de Barcelona, Parc Taulí 1, 08028 Sabadell, Barcelona Spain; 4 Clinical Pharmacology Department, Research Institute Puerta de Hierro, C/Manuel de Falla, 1, 28222 Majadahonda, Madrid Spain; 5 Servei de Farmàcia, Institut d’Investigació i Innovació Parc Taulí I3PT, Parc Taulí Hospital Universitari, Universitat Autònoma de Barcelona, Parc Taulí 1, 08028 Sabadell, Barcelona Spain; 6 Biostatistics Unit, Faculty of Medicine, Universitat Autònoma de Barcelona, 08193 Bellaterra, Barcelona Spain; 7 Medical Statistics Core Facility, IDIBAPS - Hospital Clinic Barcelona, C/Mallorca 183, Floor -1, 08036 Barcelona, Spain; 8 Unitat d’Estudis del Medicament, Institut D’Investigació en Atenció Primària IDIAP- Jordi Gol, C/Gran Via Corts Catalanes, 587, 08007 Barcelona, Spain; 9 Departament de Farmacologia, de Terapèutica i de Toxicologia, Universitat Autònoma de Barcelona, 08193 Bellaterra, Barcelona Spain; 10 Section for Medical Statistics, Center for Medical Statistics, Informatics, and Intelligent Systems, Medical University of Vienna, Spitalgasse 23, 1090 Vienna, Austria; 11 Hannover Medical School, Carl-Neuberg-Str. 1, 30625 Hannover, Germany; 12 Clinical Trial Methodology, Julius Center for Health Sciences and Primary Care, Biostatistics and Research Support, University Medical Center Utrecht, University of Utrecht, Heidelberglaan 100, 3584 CX Utrecht, The Netherlands

**Keywords:** Orphan drug production, Rare diseases, Research design/methods, Research design/standards, Clinical trials as topic, Drug approval

## Abstract

**Background:**

To assess uncertainty in regulatory decision-making for orphan medicinal products (OMP), a summary of the current basis for approval is required; a systematic grouping of medical conditions may be useful in summarizing information and issuing recommendations for practice.

**Methods:**

A grouping of medical conditions with similar characteristics regarding the potential applicability of methods and designs was created using a consensus approach. The 125 dossiers for authorised OMP published between 1999 and 2014 on the EMA webpage were grouped accordingly and data was extracted from European Public Assessment Reports (EPARs) to assess the extent and robustness of the pivotal evidence supporting regulatory decisions.

**Results:**

88% (110/125) of OMP authorizations were based on clinical trials, with 35% (38/110) including replicated pivotal trials. The mean (SD) number of pivotal trials per indication was 1.4 (0.7), and the EPARs included a median of three additional non-pivotal supportive studies. 10% of OMPs (13/125) were authorised despite only negative pivotal trials. One-third of trials (53/159) did not include a control arm, one-third (50/159) did not use randomisation, half the trials (75/159) were open-label and 75% (119/159) used intermediate or surrogate variables as the main outcome. Chronic progressive conditions led by multiple system/organs, conditions with single acute episodes and progressive conditions led by one organ/system were the groups where the evidence deviated most from conventional standards. Conditions with recurrent acute episodes had the most robust datasets. The overall size of the exposed population at the time of authorisation of OMP − mean(SD) 190.5 (202.5) − was lower than that required for the qualification of clinically-relevant adverse reactions.

**Conclusions:**

The regulatory evidence supporting OMP authorization showed substantial uncertainties, including weak protection against errors, substantial use of designs unsuited for conclusions on causality, use of intermediate variables, lack of *a priorism* and insufficient safety data to quantify risks of relevant magnitude. Grouping medical conditions based on clinical features and their methodological requirements may facilitate specific methodological and regulatory recommendations for the study of OMP to strengthen the evidence base.

**Electronic supplementary material:**

The online version of this article (10.1186/s13023-018-0926-z) contains supplementary material, which is available to authorized users.

## Background

European legislation states that market access for new drugs requires the same level of evidence, regardless of whether the drug is intended for rare or highly-prevalent diseases [1]. However, generating robust evidence with small subject samples is a methodological and logistic challenge [2] that may discourage sponsors from researching new treatments for rare diseases [3–6]. In addition, reports have warned of the potential risks of approving medicinal products when decision-making is based on limited data [4, 7–14].

Regulators prefer conventional trials to new designs because the benefit is generally read as less uncertain and they include larger pre-marketing safety populations and allow a better benefit-risk assessment and more confident decision-making. There are various reviews of the amount and quality of evidence supporting regulatory decisions on medicinal products intended for rare diseases or orphan indications -orphan medicinal products (OMP) under European regulations [1, 15–18] and of the potential risks of accelerated approval procedures when decision-making is based on limited data obtained using conventional methods [7–12].

Methodologies aiming to increase the statistical efficiency of clinical studies that might be useful in small populations have been proposed, but have mostly been applied to the clinical development of prevalent diseases, rather than rare diseases [19]. The reasons why such models are not applied to rare diseases may include the lack of predictability of regulatory requirements and sponsors’ fears of regulatory reluctance to accept non-standard methods.

Methodological guidance specific to clinical investigation of a particular disease is an effective method of providing a predictable decision-making framework [20], and is useful for developers and regulators. Such regulatory guidance for the clinical development of new medicinal products has been issued for many prevalent diseases for decades by the European Medicines Agency (EMA) [21], Food and Drug Administration (FDA) [22] and other regulatory agencies. However, there is limited disease- or medical condition-specific regulatory guidance on orphan and rare conditions: The EMA has issued two general guidance papers on small populations [23] and paediatric development [24], respectively. These provide general considerations on the rationale of regulatory assessments and the specificities of diseases that should be taken into account when tailoring clinical development to a specific clinical condition. In addition, some disease-specific documents have been issued, but for only 14 of the thousands of orphan medical conditions described [25]. The huge number of rare diseases hinders the development of disease-specific scientific, methodological, statistical and/or regulatory guidance, which would be time and resource consuming, but may not be necessary, as many diseases or situations have common features that may allow similar recommendations to be applied to their study.

From a regulatory and clinical development point of view, it may not be appropriate to refer to diseases, as defined by available medical classifications, to identify situations for which similar recommendations could be given, since the clinical development of OMP for a given disease is likely to depend also on the therapeutic approach, expected outcomes and feasible measurements, amongst other characteristics, and may differ substantially depending on the intended therapeutic indication. Thus, one disease may encompass different situations depending on the therapeutic indication (i.e. an acute infection in a patient with congenital immunodeficiency is a single acute episode with a short treatment and short time to outcome, but the underlying immune suppression is a chronic disease resulting from an underlying genetic defect requiring a permanent solution or life-lasting treatment), so that the study of each indication may require distinct methodological approaches. Thus, it may be better to talk of medical conditions resulting from the combination of disease and therapeutic indication for a given product rather than diseases.

The key first step towards improvement is to describe the current regulatory basis for approval of OMP and identify potential areas for improvement in the robustness of the data supporting regulatory decisions. In addition, knowing the reference standard is required to explore the potential impact of new statistical methods, such as those arising from the ASTERIX project [26], on the overall process of development and regulatory decision-making. Identifying uncertainties at the time of regulatory decision-making on OMP will help to focus on areas where greater robustness of the data obtained during clinical development is mainly required.

Rare diseases have in common a low prevalence but are otherwise widely heterogeneous clinically. We therefore aimed to propose a grouping of medical conditions that was sound from a regulatory and methodological perspective and could facilitate the selection of examples for testing the applicability of new methodologies. Accordingly, we developed a clustering based on medical conditions, as defined by two principle features: (i) the clinical disease and therapeutic approach or intended indication to be claimed by the OMP, and (ii) the characteristics of the condition driving requirements for the applicability of different methodologies and designs of clinical studies.

The aim of this study is to summarise the reference of the current regulatory basis for approval of OMP by the EMA, as systematized using a clustering of medical conditions, and to provide proposals for the management of the uncertainties identified and areas for improvement.

## Methods

### Development of the clustering framework

Three steps were used to build the clustering of medical conditions. First, initial clustering was made using an unsupervised statistical method −multiple correspondence analysis (MCA) [27–29] − based on potentially-informative criteria (clinical characteristics, treatment of interest, endpoints and variables, feasibility of recruitment, available treatments and treatment targets) for a representative differential set of 27 medical conditions. Secondly, the clustering was interpreted and refined by consensus between experts from different fields (regulatory, statistics, clinical). Thirdly, the clustering was validated in a larger, comprehensive set of orphan medical conditions and by an external panel of clinicians, methodologists and regulators.

The larger set of conditions consisted of all authorised OMP for which there are European Public Assessment Reports (EPAR) on the EMA webpage [30] since the inception of the Orphan Act until December 2014, and with active OMP designation at the time of authorisation (*N* = 125). The unit analysed was the EPAR, meaning the binomy OMP-medical indication as the unit assessed in the regulatory evaluation; the orphan medical indication is referred from now on as “medical condition”.

The overall process was carried out by 12 investigators with different backgrounds and expertise (public and industry drug development, medical research, statistics, medical practice, regulation, reimbursement and patient networking), with the involvement of a panel of additional external experts in the last phase.

### Development of the reference on regulatory basis for approval of OMP

The pivotal evidence supporting approval of the 125 OMP with marketing authorisations was extracted using variables describing the methods and key results of the dataset summarised in the EPAR (Additional file 1*:* Table S1). The data was analysed descriptively to identify the areas where regulatory decision-making deviated from the usually-accepted standards (i.e. statistically-significant and clinically-relevant demonstration of efficacy obtained from two replicate well-designed clinical trials [31], and a safety database compliant with ICH E1 standards [32], and to describe areas of regulatory uncertainty. Only trials identified or referenced as pivotal in the EPAR were analysed (generally phase III or phase II trials), since these are the trials supporting the risk/benefit assessment. The analysis was systematized according to six clusters of medical conditions for which the OMP applied for marketing authorisation. Prevalences were extracted from OMP designations.

Frequencies and percentages (n (%)) were used to describe qualitative variables, and mean (SD) or median (P25-P75) for quantitative variables, as appropriate.

## Results

A total of 125 EPARs were analysed that included positive opinions for 98 different active substances (14 active substances had > 1 authorised orphan indication, with a maximum of 4) authorised in 84 different orphan medical indications (20 orphan indications had positive opinions for > 1 OMP, maximum of 7).

### Clustering of medical conditions

**Fig. 1:**
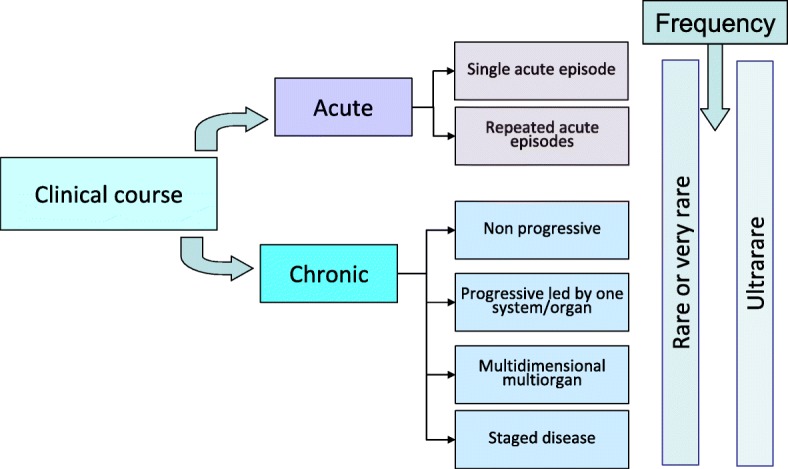
Proposed clusters of conditions

The process of clustering converged, resulting in six clusters: (1) conditions with single acute episodes, (2) conditions with recurrent acute episodes, (3) chronic slow or non-progressive conditions, (4) progressive conditions led by one organ-system, (5) progressive multidimensional conditions and (6) chronic staged conditions. The prevalence of the condition (rare: ≤5/10,000 and > 1/100,000 and ultrarare: ≤1/100,000) was taken into account due to potential implications of the limited feasibility of certain types of design and the implications for regulatory assessment [33] (Fig. 1 and Table 1).

**Table 1:** ASTERIX clustering of orphan medical conditions

Cluster	Description	Example medical conditions
(1) Conditions with single acute episode	Incident cases with single acute episode, with rapid onset and rapid endpoint: longer recruitment (incident cases) than follow-up for a given subject. Well-known and predictable course in absence of treatment, often a serious or life-threatening event. The condition is serious because of lack of effective standard of care. Recovery generally returns to baseline health status with or without sequels. Generally led by one organ/system that may develop to multiorgan impairment.	Acute leukaemias Treatment of acute angioedema Closure of patent ductus arteriosus Primary apnoea of premature newborns Treatment of anthracycline extravasations^a^
(2) Conditions with recurrent acute episodes	Prevalent conditions with clear-cut repeated episodes separated by relatively healthy periods. The condition often has a known predictable clinical course. Baseline status may deteriorate over time due to repeated episodes. The number and severity of the episodes is relevant for assessment of disease activity and subject well-being. May include two different indications: treatment of acute episodes and prevention of new episodes.	Cystic fibrosis: acute lung infection Narcolepsy Sickle cell sindrome Systemic sclerosis (scleroderma) Cryopirin-associated periodic syndromes^a^
(3) Chronic conditions with stable or slow progression	Chronic conditions that are generally life-long and affecting mainly a single function or system/organ, often due to a single deficiency or impairment, which may be inherited. The condition is relatively mild or has an acceptable standard of care that converts a serious condition into a mild condition. The clinical course is often predictable and well-known, and relatively stable so that it does not rapidly deteriorate the subject’s function or life-expectancy. However, if the standard treatment is not optimal, further deterioration may occur over time. Prevalence is higher than incidence, so there is short recruitment duration as regards to subject follow-up.	Acromegaly Essential thrombocythaemia Short bowel sindrome Chronic iron overload Lipoprotein lipase deficiency^a^
(4) Chronic progressive conditions led by one system/organ	Chronic progressively-worsening conditions where main impairment is led by one system/organ, which may or not involve others over time. The condition progressively reduces quality and/or quantity of life, typically subjects are seriously disabled due to disease. Clinical course is longer than acute conditions, usually year(s). May include inherited defects. Prevalent cases may be identified from registries where available. Current standard of care is symptomatic or supportive, but not curative. Frequent heterogeneity in clinical expression and poor predictability of clinical course.	Atypical haemolytic uremic syndrome Chronic myeloid leukaemia Cystic fibrosis: treatment of lung function decline Duchenne muscular dystrophy Inborn errors of primary bile acid synthesis^a^
(5) Chronic progressive conditions led by multiple system/organs	Life-lasting diseases, often inherited defects of metabolism that may start as paediatric conditions or in (young) adults. Often the standard of care is poor or not available. Many are ultrarare conditions. Prevalent cases may be identified from registries where available. May have highly variable clinical course, with impact on multiple system/organs, requiring multidimensional assessment and endpoints relying on subjective assessments from caregivers/patients on clinical or functional status and quality of life.	Cystic fibrosis: receptor estabilization Tuberous sclerosis Castleman’s disease Mucopolysaccharidosis^a^ Other lysosomal storage diseases^a^
(6) Chronic staged conditions	The condition initially is limited to one system/organ and then progresses/expands to life-threatening multi system/organ impairment, with clearly defined clinical stages, as defined by disease extension, each with different prognosis and different standards of care. Malignancies are generally included in this cluster. Staging is determined by disease extension or burden as measured directly by imaging, or indirectly by function as a surrogate for extension. The condition may evolve either to progression, stagnation or reversal of the condition, with time in each stage as a relevant measure of disease.	Familial Adenomatous Polyposis Pulmonary arterial hypertension Thyroid cancer, other rare solid cancers Myelodysplastic syndromes Multiple myeloma

^a^ultrarare medical conditions (prevalence < 1/100,000)

Please refer also to Additional file 1: Table S2

Eighty-five medical conditions (pairs of diseases with their corresponding therapeutic indications) were identified from the 125 EPARs published between 1999 and 2014. All medical conditions were uniquely assigned to one cluster (Additional file 1*:* Table S2)*.* EPAR for staged conditions were the largest cluster (38/125, 30%), and EPAR for conditions with recurrent acute episodes the smallest (9/125, 7%).

### Regulatory standard

Fifteen (15/125, 12%) OMP authorisations were granted in the absence of evidence from clinical trials; of these, nine were based on literature reports summarising the clinical experience on well-established use of products that had been available for many years as compounded medication or off-label used medicinal products, four were based on observational retrospective studies collecting data on clinical practice with the OMP, and two on data from compassionate programs. Thus, 110 applications were based on clinical trials (Table 2).

**Table 2:** Description of European Public Assessment Reports (EPARs) of orphan medicinal products

	Conditions with single acute episodes	Conditions with recurrent acute episodes	Chronic conditions with stable or slow progression	Chronic progressive conditions led by one system/organ	Chronic progressive conditions led by multiple system/organs	Chronic staged conditions	Total
No of EPARs	*n* = 23	*n* = 9	*n* = 13	*n* = 19	*n* = 23	*n* = 38	*n* = 125
Ultra rare condition (< 1/100.000)	1 (4.3%)	2 (22.2%)	1 (7.1%)	3 (16.7%)	9 (39.1%)	0 (0.0%)	16 (12.8%)
Type of evidence supporting the MAA approval							
Bibliographic report	3 (13.0%)	1 (11.1%)	2 (14.3%)	1 (5.6%)	1 (4.3%)	1 (2.6%)	9 (7.2%)
Compassionate use	0 (0.0%)	0 (0.0%)	0 (0.0%)	1 (5.6%)	1 (4.3%)	0 (0.0%)	2 (1.6%)
Observational retrospective	0 (0.0%)	0 (0.0%)	0 (0.0%)	0 (0.0%)	4 (17.4%)	0 (0.0%)	4 (3.2%)
Clinical trial	20 (87.0%)	8 (88.9%)	12 (85.7%)	16 (88.9%)	17 (73.9%)	37 (97.4%)	110 (88.0%)
N of MAA based on clinical trials	*n* = 20	*n* = 8	*n* = 12	*n* = 16	*n* = 17	*n* = 37	*n* = 110
> = 2 clinical trials	7 (35.0%)	5 (62.5%)	9 (75.0%)	6 (37.5%)	4 (23.5%)	7 (18.9%)	38 (34.5%)
No. of pivotal clinical trials							
Mean (SD)	1.4 (0.5)	1.6 (0.5)	2.3 (1.1)	1.6 (1.1)	1.2 (0.4)	1.2 (0.5)	1.4 (0.7)
Median (P25-P75)	1.0 (1.0–2.0)	2.0 (1.0–2.0)	2.0 (1.5–3.0)	1.0 (1.0–2.0)	1.0 (1.0–1.0)	1.0 (1.0–1.0)	1.0 (1.0–2.0)
No. of supportive trials							
Mean (SD)	3.6 (3.3)	4.3 (3.5)	3.7 (2.2)	2.2 (1.6)	2.9 (1.5)	2.9 (1.7)	3.1 (2.3)
Median (P25-P75)	3.0 (1.0–5.0)	2.5 (2.0–6.5)	3.0 (2.0–5.0)	1.5 (1.0–3.0)	2.0 (2.0–4.0)	3.0 (2.0–3.0)	3.0 (2.0–4.0)
MAA based on negative trials							
All trials negative	3 (15.0%)	1 (12.5%)	2 (16.7%)	2 (12.5%)	3 (17.6%)	2 (5.4%)	13 (11.8%)
Negative trials, at least one positive	1 (5.0%)	0 (0.0%)	2 (16.7%)	1 (6.3%)	0 (0.0%)	2 (5.4%)	6 (5.5%)
No negative trials	16 (80.0%)	7 (87.5%)	8 (66.7%)	13 (81.3%)	14 (82.4%)	33 (89.2%)	91 (82.7%)
No of trials	*n* = 27	*n* = 13	*n* = 27	*n* = 26	*n* = 21	*n* = 45	*n* = 159
Fulfilment of main study objective							
Main end-point met	23 (85.2%)	11 (84.6%)	21 (77.8%)	23 (88.5%)	18 (85.7%)	40 (88.9%)	136 (85.5%)
Not fulfilling objective	4 (14.8%)	2 (15.4%)	5 (18.5%)	2 (7.7%)	2 (9.5%)	5 (11.1%)	20 (12.6%)
Unknown	0 (0.0%)	0 (0.0%)	1 (3.7%)	1 (3.8%)	1 (4.8%)	0 (0.0%)	3 (1.9%)
Conclusion of trial based on subgroups^a^	5 (18.5%)	0 (0.0%)	1 (3.7%)	8 (30.8%)	2 (9.5%)	4 (8.9%)	20 (12.6%)
Blinding							
Double blind	8 (29.6%)	12 (92.3%)	12 (44.4%)	7 (26.9%)	15 (71.4%)	26 (57.8%)	80 (50.3%)
Single blind	1 (3.7%)	0 (0.0%)	0 (0.0%)	0 (0.0%)	0 (0.0%)	1 (2.2%)	2 (1.3%)
Open label	18 (66.7%)	1 (7.7%)	13 (48.1%)	19 (73.1%)	6 (28.6%)	18 (40.0%)	75 (47.2%)
NA	0 (0.0%)	0 (0.0%)	2 (7.4%)	0 (0.0%)	0 (0.0%)	0 (0.0%)	2 (1.3%)
Randomisation and controls							
Randomized	14 (51.9%)	13 (100.0%)	17 (63.0%)	10 (38.5%)	18 (85.7%)	37 (82.2%)	109 (68.6%)
Placebo controlled	7 (25.9%)	12 (92.3%)	12 (44.4%)	5 (19.2%)	14 (66.7%)	25 (55.6%)	75 (47.2%)
Active controlled	3 (11.1%)	1 (7.7%)	4 (14.8%)	3 (11.5%)	2 (9.5%)	8 (17.8%)	21 (13.2%)
Not controlled	12 (44.4%)	0 (0.0%)	11 (40.7%)	17 (65.4%)	4 (19.0%)	9 (20.0%)	53 (33.3%)
Other	5 (18.5%)	0 (0.0%)	0 (0.0%)	1 (3.8%)	1 (4.8%)	3 (6.7%)	10 (6.3%)
No. of Arms							
1 arm	12 (44.4%)	0 (0.0%)	8 (29.6%)	16 (61.5%)	3 (14.3%)	8 (17.8%)	47 (29.6%)
2 arms	14 (51.9%)	11 (84.6%)	14 (51.9%)	9 (34.6%)	16 (76.2%)	20 (44.4%)	84 (52.8%)
3 arms	1 (3.7%)	0 (0.0%)	4 (14.8%)	1 (3.8%)	2 (9.5%)	14 (31.1%)	22 (13.8%)
4 arms	0 (0.0%)	2 (15.4%)	1 (3.7%)	0 (0.0%)	0 (0.0%)	3 (6.7%)	6 (3.8%)
General design							
Parallel groups	14 (51.9%)	11 (84.6%)	17 (63.0%)	10 (38.5%)	17 (81.0%)	37 (82.2%)	106 (66.7%)
Single arm	12 (44.4%)	0 (0.0%)	8 (29.6%)	16 (61.5%)	3 (14.3%)	8 (17.8%)	47 (29.6%)
Crossover	0 (0.0%)	0 (0.0%)	2 (7.4%)	0 (0.0%)	1 (4.8%)	0 (0.0%)	3 (1.9%)
Randomised withdrawal	0 (0.0%)	2 (15.4%)	0 (0.0%)	0 (0.0%)	0 (0.0%)	0 (0.0%)	2 (1.3%)
Historical control	1 (3.7%)	0 (0.0%)	0 (0.0%)	0 (0.0%)	0 (0.0%)	0 (0.0%)	1 (0.6%)
Outcomes							
Final variable	12 (44.4%)	11 (84.6%)	3 (11.1%)	1 (3.8%)	4 (19.0%)	9 (20.0%)	40 (25.2%)
Intermediate variable	15 (55.6%)	2 (15.4%)	24 (88.9%)	25 (96.2%)	17 (81.0%)	36 (80.0%)	119 (74.8%)
Single variable	21 (77.8%)	11 (84.6%)	21 (77.8%)	21 (80.8%)	17 (81.0%)	31 (68.9%)	122 (76.7%)
Composite variable	1 (3.7%)	0 (0.0%)	0 (0.0%)	0 (0.0%)	1 (4.8%)	13 (28.9%)	15 (9.4%)
Co-primary variables	2 (7.4%)	2 (15.4%)	2 (7.4%)	4 (15.4%)	2 (9.5%)	1 (2.2%)	13 (8.2%)
Multiple end-points	3 (11.1%)	0 (0.0%)	4 (14.8%)	1 (3.8%)	1 (4.8%)	0 (0.0%)	9 (5.7%)
Type of variables for main outcome							
Continuous	0 (0.0%)	8 (61.5%)	11 (40.7%)	5 (19.2%)	13 (61.9%)	13 (28.9%)	50 (31.4%)
Discrete	20 (74.1%)	3 (23.1%)	13 (48.1%)	18 (69.2%)	6 (28.6%)	11 (24.4%)	71 (44.7%)
Continuous and discrete	1 (3.7%)	1 (7.7%)	3 (11.1%)	2 (7.7%)	1 (4.8%)	0 (0.0%)	8 (5.0%)
Time to event	6 (22.2%)	1 (7.7%)	0 (0.0%)	1 (3.8%)	1 (4.8%)	21 (46.7%)	30 (18.9%)
Includes biomarkers	18 (66.7%)	3 (23.1%)	22 (81.5%)	24 (92.3%)	15 (71.4%)	28 (62.2%)	110 (69.2%)
Type of objective							
Superiority	13 (48.1%)	12 (92.3%)	14 (51.9%)	8 (30.8%)	16 (76.2%)	36 (80.0%)	99 (62.3%)
Value estimation	12 (44.4%)	0 (0.0%)	11 (40.7%)	18 (69.2%)	3 (14.3%)	9 (20.0%)	53 (33.3%)
Non-inferiority	1 (3.7%)	1 (7.7%)	1 (3.7%)	0 (0.0%)	2 (9.5%)	0 (0.0%)	5 (3.1%)
NA	1 (3.7%)	0 (0.0%)	1 (3.7%)	0 (0.0%)	0 (0.0%)	0 (0.0%)	2 (1.3%)
Extent of exposure for safety assessment from pivotal trials	*n* = 20	*n* = 8	*n* = 12	*n* = 16	*n* = 17	*n* = 37	*n* = 110
Population randomized							
Mean (SD)	223.1 (177.0)	226.9 (213.3)	362.8 (408.9)	312.3 (289.0)	78.6 (57.9)	394.6 (260.5)	286.9 (268.5)
Median (P25-P75)	132.5 (95.5–386.5)	165.0 (56.0–343.5)	167.5 (120.0–571.0)	185.0 (90.5–543.5)	45.0 (39.0–118.0)	358.0 (203.0–602.0)	172.0 (87.0–447.0)
{min,Max}	{36.0–600.0}	{31.0–655.0}	{27.0–1485.0}	{12.0–1027.0}	{28.0–219.0}	{7.0–1020.0}	{7.0–1485.0}
Population on experimental treatment							
Mean (SD)	140.7 (110.9)	130.6 (124.0)	277.3 (394.3)	263.2 (272.8)	48.8 (31.7)	233.9 (133.9)	189.8 (203.0)
Median (P25-P75)	93.0 (64.0–193.0)	101.0 (28.5–197.0)	140.0 (97.0–301.5)	172.0 (45.5–416.5)	39.0 (22.0–65.0)	208.0 (125.0–318.0)	121.0 (62.0–286.0)
{min,Max}	{36.0–449.0}	{15.0–377.0}	{27.0–1485.0}	{12.0–1027.0}	{17.0–117.0}	{7.0–568.0}	{7.0–1485.0}
Safety set							
Mean (SD)	140.4 (110.3)	144.4 (127.1)	272.3 (396.5)	274.6 (270.5)	48.7 (31.6)	229.9 (131.7)	190.5 (202.5)
Median (P25-P75)	92.0 (64.0–190.0)	115.5 (34.5–239.0)	138.5 (73.5–298.5)	202.5 (44.5–421.0)	39.0 (22.0–65.0)	207.0 (124.0–300.0)	120.5 (62.0–293.0)
{min,Max}	{36.0–449.0}	{23.0–354.0}	{27.0–1485.0}	{12.0–1027.0}	{17.0–117.0}	{7.0–563.0}	{7.0–1485.0}
Rare or very rare conditions^b^	*n* = 19	*n* = 6	*n* = 11	*n* = 15	*n* = 12	*n* = 37	*n* = 100
Population randomized							
Mean (SD)	230.6 (178.5)	289.5 (211.7)	393.3 (414.3)	323.3 (295.6)	88.1 (65.8)	394.6 (260.5)	309.5 (271.3)
Median (P25-P75)	134.0 (104.0–415.0)	251.5 (139.0–375.0)	170.0 (125.0–586.0)	196.0 (88.0–559.0)	61.0 (32.0–131.0)	358.0 (203.0–602.0)	205.5 (108.0–452.0)
{min,Max}	{36.0–600.0}	{65.0–655.0}	{64.0–1485.0}	{12.0–1027.0}	{28.0–219.0}	{7.0–1020.0}	{7.0–1485.0}
Population on experimental treatment							
Mean (SD)	143.8 (113.0)	167.8 (122.0)	300.0 (405.2)	270.9 (280.6)	56.2 (33.7)	233.9 (133.9)	204.3 (207.1)
Median (P25-P75)	102.0 (63.0–207.0)	153.5 (74.0–215.0)	165.0 (111.0–306.0)	196.0 (43.0–449.0)	48.0 (28.0–77.0)	208.0 (125.0–318.0)	143.0 (69.5–298.5)
{min,Max}	{36.0–449.0}	{34.0–377.0}	{64.0–1485.0}	{12.0–1027.0}	{17.0–117.0}	{7.0–568.0}	{7.0–1485.0}
Safety set							
Mean (SD)	143.5 (112.4)	182.8 (124.5)	294.6 (407.8)	283.1 (277.7)	56.1 (33.5)	229.9 (131.7)	204.9 (206.7)
Median (P25-P75)	100.0 (63.0–201.0)	168.0 (74.0–299.0)	162.0 (83.0–301.0)	257.0 (43.0–447.0)	48.0 (28.0–77.0)	207.0 (124.0–300.0)	151.0 (65.0–297.5)
{min,Max}	{36.0–449.0}	{34.0–354.0}	{62.0–1485.0}	{12.0–1027.0}	{17.0–117.0}	{7.0–563.0}	{7.0–1485.0}
Ultrarare conditions^b^	*n* = 1	*n* = 2	*n* = 1	*n* = 1	*n* = 5	*n* = 0	*n* = 10
Population randomized							
Mean (SD)	80.0 (−)	39.0 (11.3)	27 (−)	147.0 (−)	55.8 (23.7)		61.1 (37.1)
Median (P25-P75)		39.0 (31.0–47.0)			45.0 (41.0–58.0)		46.0 (39.0–80.0)
{min,Max}		{31.0–47.0}			{39.0–96.0}		{27.0–147.0}
Population on experimental treatment							
Mean (SD)	80.0 (−)	19.0 (5.7)	27 (−)	147.0 (−)	31.0 (18.8)		44.7 (41.8)
Median (P25-P75)		19.0 (15.0–23.0)			22.0 (21.0–29.0)		25.0 (21.0–64.0)
{min,Max}		{15.0–23.0}			{19.0–64.0}		{15.0–147.0}
Safety set							
Mean (SD)	80.0 (−)	29.0 (8.5)	27 (−)	147.0 (−)	31.0 (18.8)		46.7 (40.7)
Median (P25-P75)		29.0 (23.0–35.0)			22.0 (21.0–29.0)		28.0 (22.0–64.0)
{min,Max}		{23.0–35.0}			{19.0–64.0}		{19.0–147.0}

^a^Conclusion of trial based on subgroups means granting or restriction due to positive or negative effects in subgroups

^b^Rare or very rare conditions: prevalence between ≤5/10,000 and > 1/100,000; Ultrarare: prevalence ≤1/100,000

*EPAR* European Public Assessment Report, *MAA* Marketing Authorisation Application, *SD* Standard Deviation, *min* minimum, *Max* Maximum, *P25-P75* 25th and 75Th percentiles, *NA* Not Available

The 110 OMP authorisations based on clinical trials included a total of 159 pivotal clinical trials. The mean (SD) number of pivotal trials per marketing authorisation application (MAA) was 1.4 (0.7): 38 applications were based on ≥2 pivotal trials (35% of MAA based on clinical trials, 30% of all MAA of OMP). Applications for chronic conditions with stable or slow progression had the highest mean number of pivotal trials, and applications for chronic progressive conditions led by multiple system/organs and chronic staged conditions the smallest. In addition to pivotal trials, a mean of ≥2 supportive trials were included in MAA in all clusters, with conditions with recurrent acute episodes having > 4 supportive trials per MAA.

Twenty (12.6%) pivotal trials did not fulfil the main study objective. The highest proportion of positive trials was for chronic staged conditions, whilst one third of pivotal trials on chronic conditions with stable or slow progression did not meet the main end-point. Thirteen MAA (11.8%) of those based on evidence from clinical trials did not include any pivotal trial fulfilling its main objective. Chronic staged conditions had the lowest proportion of authorisations based only on negative trials. The conclusions of 20 (12.5%) pivotal trials were based on analysis of subgroups; this represented 18/110 (16.3%) of MAA based on clinical trials; of these, 13 were predefined and five were decided *post-hoc*.

Half the pivotal clinical trials in MAA were double-blinded, ranging from 92.3% of trials for conditions with recurrent acute episodes to 26.9% for chronic progressive conditions led by one system/organ. Randomisation was applied in all pivotal trials for conditions with recurrent acute episodes and 86% for chronic progressive conditions led by multiple system/organs, but only to 38.5% for chronic progressive conditions led by one system/organ and 52% for conditions with single acute episodes. Placebo controls were used in 92.3% of trials for conditions with recurrent acute episodes but only in 19.2% of trials for chronic progressive conditions led by one system/organ and 25.9% for conditions with single acute episodes. Active controls were used in < 20% of trials in all clusters. Single arm trials were the most frequent design in chronic progressive conditions led by one system/organ (61.5%), and frequently used in conditions with single acute episodes (44.4%), while two trial arms were more frequent in conditions with recurrent acute episodes (84.6%) and chronic progressive conditions led by multiple system/organs (76.2%); three or more trial arms were relevantly used only in chronic staged conditions (37.8%). Parallel design was the most frequent setting for comparative trials. Crossover or other methods were infrequent.

Most trials in clusters for chronic conditions used intermediate primary variables; only conditions with recurrent acute episodes used mainly clinical variables as primary outcome (84.6% of trials). Discrete primary variables were used more frequently in clusters of conditions with single acute episodes and chronic progressive conditions led by one system/organ (74.1% and 69.2% of trials, respectively). Continuous variables were frequently used for trials of chronic progressive conditions led by multiple system/organs and conditions with recurrent acute episodes (61.9% and 61.5% of trials, respectively). Time variables were used frequently (46.7%) for chronic staged conditions. Chronic conditions with stable or slow progression had the highest proportion of trials with multiple primary endpoints (14.8%). Most trials had a superiority objective, but in 69.2% of trials in the cluster of chronic progressive conditions led by one system/organ the objective was to estimate value.

The size of the safety population (number of patients exposed to the product) was lower for ultra-rare conditions [median (IQR): 28 (22–64)], than for rare or very rare conditions [median (IQR): 151 (65–298)]. The cluster of progressive multidimensional conditions included the most ultrarare conditions (5/10) and also had the smallest datasets.

The uncertainties derived from the analysis of the data supporting OMP regulatory approval are summarised in Table 3.

**Table 3:** Regulatory uncertainties identified

Uncertainty	Description	Affecting mainly
Lack of clinical trials in MAA	*N* = 15 (12% of all MAA) not including clinical trials, but based on bibliographic reports, observational retrospective studies or compassionate programs. Case-series are not conclusive on causality or size of the effect; safety information was collected in a non-systematic way, making it difficult to quantify risks.	Chronic progressive conditions led by multiple system/organs Conditions with single acute episodes
Lack of 2 pivotal trials in MAA	*N* = 87 EPARs (70% of all MAA) not based on at least 2 pivotal trials. The control of type 1 error that is achieved by replication of experiments was lacking.	Chronic staged conditions Chronic progressive conditions led by multiple system/organs
Negative trials as the only basis for pivotal regulatory assessment	*N* = 13 EPARs (11.8% of MAA based on clinical trials) based on negative trials as the only basis for assessment. Risk of approval of ineffective therapies based on insufficient evidence.	Chronic progressive multidimensional conditions Acute single episodes
Low level of evidence of pivotal data	Pivotal clinical trials in MAA using open label (*N* = 75, 47.2% of all pivotal trials), non-randomised (*N* = 50, 31.4% of all pivotal trials) and/or not controlled designs (*N* = 53, 33.3% of all pivotal trials). Lack of robustness for causality assessment, risk of bias and risk of overestimation of benefits.	Conditions with single acute episodes Chronic progressive conditions led by one system/organ Chronic progressive conditions led by multiple system/organs
Use of surrogate or intermediate primary variables	*N* = 119 (74.8% of all trials) using intermediate variables as main end-point. Risk that improvements in intermediate parameters are not reliable indicators of clinical benefit, and risk of overestimation of benefits.	Chronic conditions with stable or slow progression Chronic progressive conditions led by one system/organ Chronic progressive conditions led by multiple system/organs Chronic staged conditions
Conclusions based on post-hoc analyses	20 pivotal trials (12.6% of all pivotal trials) concluded based on subgroup analysis, of which 5 were not pre-defined. Risk of type 1 error and bias due to data-guided analysis.	Chronic progressive conditions led by one system/organ Chronic progressive conditions led by multiple system/organs
Small extent of population exposure to assess clinical safety	Mean size of the available safety population smaller than recommended by ICH E1; much lower amongst ultra-rare conditions. Lack of knowledge on frequently expected events do not allow a reliable risk/benefit assessment.	Ultrarare conditions Chronic progressive conditions led by multiple system/organs

*EPAR* European Public Assessment Report, *MAA* Marketing Authorisation Application

## Discussion

### Summary of findings

We analysed the current basis for regulatory approval for OMP in the European Union (EU). The results show that 88% (110/125) of OMP authorizations were based on clinical trials, of which only 35% complied with the usual regulatory standard of ≥2 replicated pivotal trials [34]. The mean number of pivotal trials per indication was 1.45 and half the pivotal trials were phase II trials. Likewise, 13% of OMP approvals included clinical trials that did not meet their main objective, which could be considered consistent with the theoretically-expected number of false negatives in a standard scenario, but almost 10% of EPAR were authorised based only on negative trials. The overall size of the exposed population at the time of authorisation was generally lower than that required for the qualification of clinically-relevant adverse reactions [32]. Reports have described similar results concerning the number of trials and the proportion of phase III trials, but none has reported on the proportion of negative trials [35].

### Quality of scientific evidence

One-third of trials did not include a control arm, one-third did not use randomisation, half were open-label and 75% used intermediate or surrogate variables as the main outcome. These characteristics differ substantially from the recommended standards [36]. Differences between trials in orphan medical conditions compared with those in prevalent conditions have been reported, including a higher frequency of non-controlled study designs, the lesser use of randomized allocation of patients, a higher percentage of open-label trials and fewer individuals enrolled [4, 15, 16, 37, 38]. As expected, noticeably smaller sample sizes are reported for ultra-rare diseases (prevalence < 1/100,000) compared with more prevalent rare diseases (prevalence between ≥1/100,000 and 50/100,000) [39]. All these features are related to the risk of bias, and may increase type 1 error, suggesting that current evidence supporting OMP authorizations might be biased towards a higher chance of positive results [40].

Although pivotal trials generally included small numbers of patients, the EPAR included a median of three additional supportive studies (i.e.: non-pivotal trials) per authorised indication. In general, the median number of supportive trials doubled the number of pivotal trials, suggesting that the number of patients recruited into pivotal trials may potentially have been higher, meaning that bigger sample sizes might have been feasible; this would had allowed to detect smaller effects, increase power and potentially reduced the likelihood of negative trials [40]. Supportive trials were likely a relevant source of additional data to support decision-making, especially in applications including no pivotal trials, those based on one single pivotal trial and – especially – only on negative trials. Supportive studies contribute to the assessment of dose ranging, the clinical relevance of main end-points, and the duration of effects and safety issues, and are a source of complementary information in a setting of a scarcity of pivotal evidence [36]. Thus, in the context of the relative scarcity of data in OMP dossiers, supportive studies become especially relevant, and it is of utmost importance maximizing the quality of any study or research during the product development, i.e. from early proof-of-concept trials to open-label extension safety cohorts.

These findings suggest that, on the one hand, the generation of robust scientific evidence for OMP is a hard challenge and, on the other hand, that regulators are often taking decisions on OMP based on weak scientific evidence [15, 41, 42].

### Findings in clusters of medical conditions

Authorization in the absence of clinical trials was more frequent in the cluster of chronic progressive conditions led by multiple system/organs, which included many inherited diseases affecting children. There were a number of EPAR that recognised well-established uses of products already available in clinical practice, whose authorization was probably unavoidable [43]. The applications included both retrospective studies, which have a low level of robustness and are a source of uncertainty for decision-making, but also prospective registries and compassionate programs. The latter may allow structured, complete information on effectiveness and safety to be obtained, provided that the design is made considering their future utility as a source of data for priors in Bayesian designs or as an external reference [44]. However, the data is not comparative and is of small value in assessing causality [36]. Specific meta-analytical techniques can be applied to studies to ease the interpretation of data at the time of regulatory assessment [40].

Negative trials were observed across all clusters, but less frequently in conditions with recurrent acute episodes and chronic staged conditions. The clinical setting of conditions with recurrent acute episodes allows designs based on repeated measurements and paired data, both of which increase the efficiency of trials [36]. In the case of chronic staged conditions, the smaller number of negative trials might be related to an overall greater number of patients included than for other clusters, but the fact that the trials were often open-label may have also contributed [40, 45].

In 61.5% of pivotal trials for chronic progressive conditions led by one system/organ and 44.4% of those for conditions with single acute episodes the design had an inherently-low potential to conclude causality, due to lack of control and open-label designs with a single arm. Both clusters included many serious conditions with a lack of an acceptable standard of care. The willingness to provide any potential treatment (even in a scenario of huge uncertainty) for patients lacking alternatives, in response to the ethical right of beneficence, may have precluded the conduct of comparative designs [46]. In such a setting, efficacy may be overestimated for many reasons (lack of comparator, lack of blinding, use of historical controls with different background therapies and reliance on surrogate, non-validated variables based on subjective assessments, amongst others). Thus, the lack of conclusive information is a reason for concern for patients when granting regulatory approval, since there is a poor basis to determine the efficacy and safety of the new products [44].

The percentage of EPAR based on replicated trials was < 20% in the cluster of chronic progressive conditions led by multiple system/organs, which also had the lowest mean number of patients exposed. This may be because this cluster includes many ultrarare and often inherited paediatric conditions, where the feasibility of recruitment is limited and, accordingly, few subjects could potentially be recruited for (replicated) trials. In contrast, the cluster of staged conditions also had < 20% of EPAR based on replicated pivotal trials, with evidence based mainly on one (often phase II) trial, but this cluster represented mostly adult malignancies, with no ultrarare conditions, and with the highest mean number of exposed patients. This suggests that that the lack of replicated trials in this case is not related to the disease prevalence, but rather to the reduced requirements due to early access policies in the context of perceived severity and medical need. In fact, warnings on the overestimation of benefits at the time of approval under early access policies have been raised [47].

The cluster of conditions with single acute episodes had a higher proportion of decisions based on data other than clinical trials or on negative trials, taken in the absence of positive trials and lacking replicated trials, suggesting that clinical research may be especially challenging for many reasons in this cluster.

Conclusions based only on subgroup analysis were observed in 13% of trials, but in one-third of positive opinions for chronic progressive conditions led by one system/organ, and in some cases these were *post-hoc* subgroup analyses of otherwise negative trials. These conditions are characterized by a poor prognosis that makes it ethically difficult to conduct conventional controlled double-blind parallel trials, but also by substantial clinical heterogeneity. However, the EMA [48] warns against the risks of subgroup analyses potentially leading to unreliable inferences and, consequently, poor decisions, due to their increased probability of false-positive findings, especially if data-driven, and gives specific mention to the inappropriate “rescue” of negative trials through subgroup analysis. Thus, especial care should be paid to the pre-determination of subgroups in this setting.

The type of primary variables (discrete vs continuous, final vs surrogate, time to event) allowed discrimination between clusters. Clusters including chronic conditions mainly had primary variables based on surrogates; for chronic progressive conditions led by one system/organ, the variables were often functional and based on subjective assessment. While surrogates have many benefits in that they may improve trial power and the ability to describe product activity, warnings on overreliance on intermediate variables have repeatedly been made: surrogates may not actually predict clinical benefits, can mislead physicians on whether a drug works and have the potential to expose patients to poorly effective treatments or unanticipated adverse effects [4].

### Study limitations

The study had a number of limitations. First, it was based only on data from medicines approved in the EU, when they received marketing authorisation from the European Commission and had an orphan drug designation. Three groups of medicines were excluded: (a) medicines authorised before the orphan drug legislation entered into force, (b) medicines without an ODD, and (c) medicines that held an ODD during development, but not at the time of marketing authorisation. Comparisons to standards in other regions, or to decisions issued by other regulatory bodies were out of the scope of the current exercise. Secondly, regulatory evidence was analysed using only conditions for which an approved OMP was already available, and this may be regarded as a source of bias, because successful OMP may over-represent conditions for which conventional research methods are actually applicable, making new treatments easier to study and develop [38]. Partial selection of the data used to describe current practice may lead to a biased picture of the actual methods used in clinical research for OMP. However, the available information on negative opinions has only recently been published, and is less extensive than that for positive opinions [30], and there are no other publicly available sources for systematized information on the evidence supporting regulatory decisions. In addition, the description of the regulatory standard in authorised OMP showed that replicated parallel randomised double-blind trials were not the rule.

Thirdly, product labelling has been proposed as a flawed source for the study of orphan drug approvals [4]. However, EPAR include detailed information on the basis for regulatory decisions, including thorough discussion on the strengths and weaknesses of data [30]. Even so, there was heterogeneity in the extension and detail of the EPAR over time, so that the reliability of information on specific trial details, i.e. pre-definition of subgroup analysis, cannot be ensured. We may have overestimated some parameters due to a lack of details in the EPAR; similar limitations have been reported [35]. Fourthly, we did not extract details on the actual statistical methods applied (i.e. adaptations, interim analyses or type of adjustments for multiplicity). Fifthly, we compared the robustness of data supporting regulatory decisions using conventional methodological standards as a reference [36], but did not consider other aspects such as the effect size, the degree of unmet medical need or contextual considerations. Thus, the possibility that conclusions on the weakness of supportive evidence may be overestimated cannot be ruled out. However, such criteria, when mentioned in EPAR, are referred to as narrative statements under the risk-benefit considerations, not systematized, and generally referred to the singularity of cases. Due to the lack of available references on the acceptability of these criteria for robustness of data, we limited our analysis to conventional items on methodological quality. Finally, we focused our analysis on the areas of uncertainty at the time of decision making, but did not study whether uncertainty resulted later in lack of effectiveness in real life, or drug withdrawal for safety reasons; such objective was out of the scope of the current work, and would require further investigation.

The clustering proposal was built based on a limited number of conditions, which could be regarded as too small to be representative of the overall complexity of the huge number of orphan and rare conditions [25]. However, the description of the regulatory standard across the clusters showed that the EPAR included similar situations and methodological approaches to the development of OMP that were shared by several OMP within a given cluster, and is useful in identifying where the key challenges in the design and selection of outcomes for a given development in different groups of medical conditions lie.

The development of new methodologies and statistical approaches to the study of rare diseases have been boosted in recent years, in part thanks to the FP7 initiative funding three projects (ASTERIX, IDeAl, and InSPiRe) [49] on improving methods suited to the study of small populations. However, the translation of statistical advances to practice has traditionally been a challenge, because of perceived technical complexity and regulatory reluctance to deviate from the double-blind randomised gold standard. Any initiative aimed at facilitating the dissemination of methods and focused guidance may help to improve their uptake and, consequently, may facilitate better research into OMP. Such an unmet need was noted in a recent expert discussion (*Small Population Clinical Trials Task Force* led by IRDiRC [2] which agreed that a classification of rare diseases suitable to discuss the potential application of different study methods or designs was required. Our clustering proposal might be a contribution to this aim. By bridging the distance between too general guidance and unfeasible disease-specific guidance, it may help to systematize such dissemination and guidance. Our proposal differs from other medical or clinical classifications [25, 50, 51] in that the proposed clusters agglutinate rare medical conditions, rather than rare diseases, and may be a pragmatic way of identifying situations where new developments are required, and where newly developed methods could add value. Our proposal may require further validation and refining if new conditions appear that are unclassifiable but, until now have been acceptable to describe the current situation for authorised OMP in the EU, and to systematize situations where certain methodologies or study designs may be applicable in order to structure the output of the ASTERIX project.

## Conclusions

Our description of the regulatory evidence supporting OMP authorization has identified substantial uncertainties, such as weaker protection against type 1 and type 2 errors, the use of designs unsuited to conclude on causality, the use of intermediate variables without validation, a lack of *a priorism* and insufficient safety data to quantify risks of relevant magnitude. Some of these features are not exclusive to rare diseases and some may be unavoidable in some situations because of the sometimes (ultra-) rare nature of the disease. However, it is reasonable to assume that there are opportunities for improvement, including increasing the application of available methods and designs that may be more efficient or robust in small populations, but also the development of novel methods better suited to these conditions. A clustering of medical conditions based on the convergence of clinical features and their methodological requirements is proposed, aimed at facilitating the production of specific methodological and regulatory recommendations, and as a framework for the testing and validation of new methods for the study of OMP.

## Additional file

Additional file 1: **Table S1.** Characteristics used to describe the evidence supporting marketing authorization application in European Public Assessment Reports of Orphan Medicinal Products. **Table S2.** Clustering of the 85 medical conditions in European Public Assessment Reports of Orphan Medicinal Products. **Table S3.** Dictionary of some methodological terms. **Figure S1.** Number of European Public Assessment Reports in each cluster. (DOCX 47 kb)

## Abbreviations

ASTERIX: Advances in Small Trials dEsign for Regulatory Innovation and eXcellence
BMK: Biomarker
EMA: European Medicines Agency
EPAR: European Public Assessment Report
EU: European Union
FDA: Food and Drug Administration
FP7: Seventh Framework Program
ICH: International Conference on Harmonization
IDeAl: Integrated DEsign and Analysis of small population group trial
InSPiRe: Innovation in Small Populations Research
IQR: Inter Quartile Range
IRDiRC: International Rare Diseases Research Consortium
MAA: Marketing Authorisation Application
MCA: Multiple Correspondence Analysis
OMP: Orphan Medicinal Product
QoL: Quality of Life
SD: Standard Deviation
SOC: Standard Of Care

## References

[1] European Parliament C of the EU. Regulation (EC) No 141/2000 of the European Parliament and of the Council of 16 December 1999 on orphan medicinal products. Official Journal of the European Communities. https://ec.europa.eu/health/sites/health/files/files/eudralex/vol-1/reg_2000_141_cons-2009-07/reg_2000_141_cons-2009-07_en.pdf. Published 2000. Accessed 12 Oct 2018..

[2] Jonker A, Mills A, Lau L, et al., eds. Small Population Clinical Trials: Challenges in the Field of Rare Diseases. 2016. http://www.irdirc.org/wp-content/uploads/2017/12/SPCT_Report.pdf. Accessed 12 Oct 2018.

[3] Roberts SA, Allen JD, Sigal EV (2011). Despite criticism of the FDA review process, new Cancer drugs reach patients sooner in the United States than in Europe. Health Aff.

[4] Kesselheim AS, Myers JA, Avorn J (2011). Characteristics of clinical trials to support approval of orphan vs nonorphan drugs for cancer. JAMA.

[5] Jonsson B, Bergh J (2012). Hurdles in anticancer drug development from a regulatory perspective. Nat Rev Clin Oncol.

[6] Griggs RC, Batshaw M, Dunkle M (2009). Clinical research for rare disease: opportunities, challenges, and solutions. Mol Genet Metab.

[7] Apolone G, Joppi R, Bertele’ V, Garattini S (2005). Ten years of marketing approvals of anticancer drugs in Europe: regulatory policy and guidance documents need to find a balance between different pressures. Br J Cancer.

[8] Tsimberidou A-M, Braiteh F, Stewart DJ, Kurzrock R (2009). Ultimate fate of oncology drugs approved by the US Food and Drug Administration without a randomized trial. J Clin Oncol.

[9] Heemstra HE, Giezen TJ, Mantel-Teeuwisse AK, RL a d V, Leufkens HGM (2010). Safety-related regulatory actions for orphan drugs in the US and EU: a cohort study. Drug Saf.

[10] Gaddipati H, Liu K, Pariser A, Pazdur R (2012). Rare Cancer trial design: Lessons from FDA approvals. Clin Cancer Res.

[11] Arnardottir AH, Haaijer-Ruskamp FM, Straus SMJ, Eichler H-G, P a d G, Mol PGM (2011). Additional safety risk to exceptionally approved drugs in Europe?. Br J Clin Pharmacol.

[12] Niraula S, Seruga B, Ocana A (2012). The price we pay for progress: a meta-analysis of harms of newly approved anticancer drugs. J Clin Oncol.

[13] Halpern SD, Karlawish JHT, Berlin JA (2002). The continuing unethical conduct of underpowered clinical trials. JAMA.

[14] Joppi R, Bertele’ V, Garattini S (2013). Orphan drugs, orphan diseases. The first decade of orphan drug legislation in the EU. Eur J Clin Pharmacol.

[15] Dupont AG, Van Wilder PB (2011). Access to orphan drugs despite poor quality of clinical evidence. Br J Clin Pharmacol.

[16] Orfali M, Feldman L, Bhattacharjee V (2012). Raising orphans: how clinical development programs of drugs for rare and common diseases are different. Clin Pharmacol Ther.

[17] Richey EA, Lyons EA, Nebeker JR (2009). Accelerated approval of cancer drugs: improved access to therapeutic breakthroughs or early release of unsafe and ineffective drugs?. J Clin Oncol.

[18] Cheng M, Ramsey S, Devine E, Garrison L, Bresnahan B, Veenstra D (2012). Systematic review of comparative effectiveness data for oncology orphan drugs. Am J Manag Care.

[19] Gagne JJ, Thompson L, O’Keefe K, Kesselheim AS (2014). Innovative research methods for studying treatments for rare diseases: methodological review. BMJ.

[20] Roes KCB (2016). A framework: make it useful to guide and improve practice of clinical trial design in smaller populations. BMC Med.

[21] European Medicines Agency. Human regulatory, Research and development: Scientific guidelines. https://www.ema.europa.eu/human-regulatory/research-development/scientific-guidelines. Accessed 8 Jan 2018.

[22] Food and Drug Administration. Guidance Documents. http://www.fda.gov/RegulatoryInformation/Guidances/default.htm. Accessed 12 Oct 2018.

[23] European Medicines Agency. Guideline on Clinical Trials in Small Populations. CHMP/EWP/83561/2005. https://www.ema.europa.eu/en/clinical-trials-small-populations. Published 2006. Accessed 12 Oct 2018.

[24] European Medicines Agency. Draft reflection paper on the use of extrapolation in the development of medicines for paediatrics. EMA/199678/2016. 2017;44(October):1–14. https://www.ema.europa.eu/documents/scientific-guideline/reflection-paper-pharmaceutical-development-medicines-use-older-population-first-version_en.pdf. Accessed 12 Oct 2018

[25] Orphanet. The portal for rare diseases and orphan drugs. https://www.orpha.net/consor/cgi-bin/index.php. Accessed 12 Oct 2018.

[26] ASTERIX: Advances in Small Trials dEsign for Regulatory Innovation and eXcellence, FP7 HEALTH 2013–603160. http://www.asterix-fp7.eu. Accessed 8 Jan 2018.

[27] Benzécri J, Bellier L (1973). L’analyse Des Données, Tome 2: L’analyse Des Correspondances. 1ère.

[28] Greenacre MJ (1983). Theory and applications of correspondence analysis.

[29] Greenacre M (1992). Correspondence analysis in medical research. Stat Methods Med Res.

[30] European Medicines Agency. European Public Assessment Reports (EPARs). https://www.ema.europa.eu/en/about-us/how-we-work/what-we-publish/european-public-assessment-reports. Accessed 12 Oct 2018.

[31] International Conference on Harmonization. Integrated addendum toICH E6(R1): Guideline for Good Clinical Practice E6(R2). *ICH Guidel*. 2016;6(November):66. http://www.ich.org/fileadmin/Public_Web_Site/ICH_Products/Guidelines/Efficacy/E6/E6_R2__Step_4_2016_1109.pdf. Accessed 12 Oct 2018.

[32] International Conference on Harmonisation. ICH Harmonised Tripartite Guideline. The Extent of Population Exposure to Assess Clinical Safety for Drugs Intended for Long-Term Treatment of Non-Life-Threatening Conditions E1. http://www.ich.org/fileadmin/Public_Web_Site/ICH_Products/Guidelines/Efficacy/E1/Step4/E1_Guideline.pdf. Published 1994. Accessed 17 Jan 2018.

[33] Maeda K, Kaneko M, Narukawa M, Arato T (2017). Points to consider: efficacy and safety evaluations in the clinical development of ultra-orphan drugs. Orphanet J Rare Dis..

[34] European Medicines Agency. Points to Consider on Application with 1. Meta-analyses; 2. One pivotal study (CPMP/EWP/2330/99). https://www.ema.europa.eu/documents/scientific-guideline/points-consider-application-1meta-analyses-2one-pivotal-study_en.pdf. Published 2001. Accessed 12 Oct 2018.

[35] Picavet E, Cassiman D, Hollak CE, J a M, Simoens S (2013). Clinical evidence for orphan medicinal products-a cause for concern?. Orphanet J Rare Dis..

[36] International Conference on Harmonization. ICH Harmonised Tripartite Guideline. Statistical Principles for Clinical Trials E9. CPMP/ICH/363/96. http://www.ich.org/fileadmin/Public_Web_Site/ICH_Products/Guidelines/Efficacy/E9/Step4/E9_Guideline.pdf. Published 1998. Accessed 24 Mar 2018.

[37] Mitsumoto J, Dorsey ER, Beck CA, Kieburtz K, Griggs RC (2009). Pivotal studies of orphan drugs approved for neurological diseases. Ann Neurol.

[38] Bell SA, Tudur Smith C (2014). A comparison of interventional clinical trials in rare versus non-rare diseases: an analysis of ClinicalTrials.gov. Orphanet J Rare Dis..

[39] Hee SW, Willis A, Tudur Smith C (2017). Does the low prevalence affect the sample size of interventional clinical trials of rare diseases? An analysis of data from the aggregate analysis of clinicaltrials.gov. Orphanet J Rare Dis..

[40] Gluud LL (2006). Bias in clinical intervention research. Am J Epidemiol.

[41] Kanters TA, de Sonneville-Koedoot C, Redekop WK, Hakkaart L (2013). Systematic review of available evidence on 11 high-priced inpatient orphan drugs. Orphanet J Rare Dis..

[42] Onakpoya IJ, Spencer EA, Thompson MJ, Heneghan CJ (2015). The effectiveness, safety and costs of orphan drugs: an evidence-based review. Clin Ther.

[43] Buckley BM (2008). Clinical trials of orphan medicines. Lancet.

[44] Califf RM (2017). Balancing the need for access with the imperative for empirical evidence of benefit and risk. JAMA.

[45] Jadad Alejandro R., Moore R.Andrew, Carroll Dawn, Jenkinson Crispin, Reynolds D.John M., Gavaghan David J., McQuay Henry J. (1996). Assessing the quality of reports of randomized clinical trials: Is blinding necessary?. Controlled Clinical Trials.

[46] de Melo-Martín I, Sondhi D, Crystal RG (2011). When ethics constrains clinical research: trial Design of Control Arms in “greater than minimal risk” pediatric trials. Hum Gene Ther.

[47] Davis C, Naci H, Gurpinar E, Poplavska E, Pinto A, Aggarwal A (2017). Availability of evidence of benefits on overall survival and quality of life of cancer drugs approved by European medicines agency: retrospective cohort study of drug approvals 2009-13. BMJ.

[48] European Medicines Agency. Guideline on the investigation of subgroups in confirmatory clinical trials (EMA/CHMP/539146/2013). https://www.ema.europa.eu/documents/scientific-guideline/draft-guideline-investigation-subgroups-confirmatory-clinical-trials_en.pdf. Published 2014. Accessed 12 Oct 2018.

[49] Hilgers R-D, Roes K, Stallard N (2016). Directions for new developments on statistical design and analysis of small population group trials. Orphanet J Rare Dis.

[50] World Health Organization. International Statistical Classification of Diseases and Related Health Problems 10th Revision. http://apps.who.int/classifications/icd10/. Accessed 8 Jan 2018.

[51] Johns Hopkins University. OMIM - Online Mendelian Inheritance in Man®: An Online Catalog of Human Genes and Genetic Disorders. http://omim.org. Accessed 8 Jan 2018.

