# Supplementary material for: Evidence supporting regulatory-decision making on orphan medicinal products authorisation in Europe: methodological uncertainties
Source: Orphanet J Rare Dis. 2018 Nov 15;13:206. doi: 10.1186/s13023-018-0926-z (PMC6238348; doi:10.1186/s13023-018-0926-z)
Supplement: Supplementary file 1 — Table S1. Characteristics used to describe the evidence supporting marketing authorization application in European Public Assessment Reports of Orphan Medicinal Products. Table S2. Clustering of the 85 medical conditions in European Public Assessment Reports of Orphan Medicinal Products. Table S3. Dictionary of some methodological terms. Figure S1. Number of European Public Assessment Reports in each cluster. (DOCX 47 kb) [file 13023_2018_926_MOESM1_ESM.docx]

**Table S1. Characteristics used to describe the evidence supporting marketing authorization application in European Public Assessment Reports of Orphan Medicinal Products**

| **Scope** | **Characteristics analysed** |
| --- | --- |
| **For each therapeutic indication approved** | - Active principle - Brand name - New medicine or Extension of therapeutic indication - Orphan medical condition - Type of scientific evidence supporting the marketing approval - Number of pivotal clinical trials conducted (if exist) - Number of supportive clinical trials conducted (if exist) - Overall number of patients exposed to the orphan medicinal product - Longest follow up time period available - ASTERIX project Identification Number |
| **For each main clinical trial being part of the application submitted to the EMA to obtain the marketing authorisation**  **(only analysed for therapeutic indications approved based on clinical trials)** | - Study name - Type of phase - Type of blinding - Existence or absence of randomization - Existence or absence of stratification in the randomization process - Type of control (if exist) - Experimental treatment used as add on treatment or not - Number of arms included in the clinical trial - Number of arms included in the clinical trial that received the experimental treatment - National or multinational trial - Mono center or multi-center trial - Overall population enrolled in the clinical trial - Population enrolled in the clinical trial whom received the experimental treatment - Type of methodological design applied in the clinical trial - Clinical trial following a Survival design (Yes/No) - Description of the primary endpoint - Duration of primary endpoint - Type of statistical analysis conducted for the primary endpoint - Description of the outcome for the primary endpoint - Fulfillment of the primary objective - Key secondary endpoints - Pre-specified subgroup analysis conducted - Marketing authorisation based on subgroups analysis - ASTERIX project Identification Number |

ASTERIX: Advances in Small Trials dEsign for Regulatory Innovation and eXcellence; EMA: European Medicines Agency

**Table S2.** Clustering of the 85 medical conditions in European Public Assessment Reports of Orphan Medicinal Products

| **Clustering** | **Ultrarare** | **Medical condition** | **Drug** | **OMP** |
| --- | --- | --- | --- | --- |
| **Conditions with Single Acute Episodes** | No | Acute lymphoblastic leukaemia | Clofarabine | Evoltra |
|  |  |  | Dasatinib | Sprycel |
|  |  |  | Imatinib | Glivec |
|  |  |  | Mercaptopurine | Xaluprine |
|  |  |  | Nelarabine | Atriance |
|  |  |  | Ponatinib | Iclusig |
|  |  | Acute myeloid leukaemia | Arsenic trioxide | Trisenox |
|  |  |  | Azacitidine | Vidaza |
|  |  |  | Decitabine | Dacogen |
|  |  |  | Histamine dihydrochloride | Ceplene |
|  |  | Angioedema | Icatibant | Firazyr |
|  |  | Hepatic veno-occlusive disease | Defibrotide | Defitelio |
|  |  | Intra-operative diagnosis of residual glioma | 5-aminolevulinic acid | Gliolan |
|  |  | Patent ductus arteriosus | Ibuprofen | Pedea |
|  |  | Primary apnoea of premature newborns | Caffeine | Peyona |
|  |  | Severe skin burns | Bromelain | NexoBrid |
|  |  | Tuberculosis | Bedaquiline | Sirturo |
|  |  |  | Delamanid | Deltyba |
|  |  |  | Para-aminosalicylic acid | Granupas |
|  |  | Conditioning for hematological transplantation | Busulfan | Busilvex |
|  |  |  | Plerixafor | Mozobil |
|  |  |  | Thiotepa | Tepadina |
|  | Yes | Anthracycline extravasations | Dexrazoxane | Savene |
| **Conditions with Recurrent Acute Episodes** | No | Cystic fibrosis: lung infection | Aztreonam | Cayston |
|  |  |  | Tobramycin | TOBI Podhaler |
|  |  | Lennox-Gastaut syndrome | Rufinamide | Inovelon |
|  |  | Narcolepsy | Sodium oxybate | Xyrem |
|  |  | Severe myoclonic epilepsy in infancy | Stiripentol | Diacomit |
|  |  | Sickle cell síndrome | Hydroxiurea | Siklos |
|  |  | Systemic sclerosis (scleroderma) | Bosentan | Tracleer |
|  | Yes | Cryopirin-associated periodic syndromes | Canakinumab | Ilaris |
|  |  |  | Rilonacept | Rilonacept |
| **Chronic Conditions with Stable or Slow Progression** | No | Acromegaly | Pasireotide | Signifor |
|  |  |  | Pegvisomant | Somavert |
|  |  | Adrenal insufficiency | Hydrocortisone | Plenadren |
|  |  | Chronic idiopathic thrombocytopenic purpura | Eltrombopag | Revolade |
|  |  |  | Romiplostim | Nplate |
|  |  | Chronic pain | Ziconotide | Prialt |
|  |  | Cushing’s síndrome | Ketoconazole | Ketoconazole HRA |
|  |  | Cushing's disease | Pasireotide | Signifor |
|  |  | Essential thrombocythaemia | Anagrelide | Xagrid |
|  |  | Lambert-Eaton myasthenic syndrome | Amifampridine | Firdapse |
|  |  | Short bowel síndrome | Teduglutide | Revestive |
|  |  | Chronic iron overload | Deferasirox | Exjade |
|  |  | Primary IGF-1 deficiency | Mecasermin | Increlex |
|  | Yes | Lipoprotein lipase deficiency | Alipogene tiparvovec | Glybera |
| **Chronic Progressive Conditions Led by One System/Organ** | No | Atypical haemolytic uremic syndrome | Eculizumab | Soliris |
|  |  | Chronic myeloid leukaemia | Bosutinib | Bosulif |
|  |  |  | Dasatinib | Sprycel |
|  |  |  | Imatinib | Glivec |
|  |  |  | Nilotinib | Tasigna |
|  |  |  | Ponatinib | Iclusig |
|  |  | Cystic fibrosis: symptomatic | Mannitol | Bronchitol |
|  |  | Dermatofibrosarcoma protuberans | Imatinib | Glivec |
|  |  | Duchenne muscular dystrophy | Ataluren | Translarna |
|  |  | Erythropoietic protoporphyria | Afamelanotide | Scenesse |
|  |  | Malignant gastrointestinal stromal tumours | Sunitinib | Sutent |
|  |  | Paroxysmal nocturnal haemoglobinuria | Eculizumab | Soliris |
|  |  | Wilson's disease | Zinc | Wilzin |
|  |  | Chronic myelofibrosis | Ruxolitinib | Jakavi |
|  |  | Chronic eosinophilic leukaemia/hypereosinophilic sd | Imatinib | Glivec |
|  | Yes | Inborn errors of primary bile acid synthesis | Cholic acid | Kolbam |
|  |  |  |  | Orphacol |
|  |  |  |  |  |
|  |  | Malignant gastrointestinal stromal tumours | Imatinib | Glivec |
| **Chronic Progressive Conditions Led by Multiple System/Organs** | No | Castleman's disease | Siltuximab | Sylvant |
|  |  | Cystic fibrosis: receptor estabilization | Ivacaftor | Kalydeco |
|  |  | Cystinosis | Mercaptamine | Procysbi |
|  |  | Familial amyloid polyneuropathy | Tafamidis | Vyndaqel |
|  |  | Gaucher disease | Miglustat | Zavesca |
|  |  |  | Velaglucerase alfa | VPRIV |
|  |  | Homocystinuria | Betaine anhydrous | Cystadane |
|  |  | Hyperphenylalaninemia | Sapropterin | Kuvan |
|  |  | Mucopolysaccharidosis type IVA | Elosulfase alfa | Vimizim |
|  |  | Niemann-Pick disease, type C | Miglustat | Zavesca |
|  |  | Tuberous sclerosis - AML | Everolimus | Votubia |
|  |  | Tuberous sclerosis - SEGA | Everolimus | Votubia |
|  |  | Tyrosinaemia type I | Nitisinone | Orfadin |
|  |  | Glycogen storage disease type II (Pompe) | Alglucosidase alfa | Myozyme |
|  | yes | Fabry disease | Agalsidase alfa | Replagal |
|  |  |  | Agalsidase beta | Fabrazyme |
|  |  | Isovaleric acidaemia | Carglumic acid | Carbaglu |
|  |  | methylmalonic acidaemia | Carglumic acid | Carbaglu |
|  |  | Mucopolysaccharidosis type I | Laronidase | Aldurazyme |
|  |  | Mucopolysaccharidosis type II | Idursulfase | Elaprase |
|  |  | Mucopolysaccharidosis type VI | Galsulfase | Naglazyme |
|  |  | N-acetylglutamate synthetase deficiency | Carglumic acid | Carbaglu |
|  |  | Propionic acidaemia | Carglumic acid | Carbaglu |
| **Chronic Staged Conditions** | No | Adrenal cortical carcinoma | Mitotane | Lysodren |
|  |  | Anaplastic large cell lymphoma | Brentuximab | Adcetris |
|  |  | Chronic lymphocytic leukaemia | Ibrutinib | Imbruvica |
|  |  |  | Obinutuzumab | Gazyvaro |
|  |  |  | Ofatumumab | Arzerra |
|  |  | Dysplasia in Barrett's Esophagus | Porfimer sodium | Photobarr |
|  |  | Familial Adenomatous Polyposis | Celecoxib | Onsenal |
|  |  | Follicular thyroid cancer/ papillary thyroid cancer | Sorafenib | Nexavar |
|  |  | Gastric cancer | Ramucirumab | Cyramza |
|  |  | Hairy cell leukaemia | Cladribine | Litak |
|  |  | Hepatocellular carcinoma | Sorafenib | Nexavar |
|  |  | Hodgkin lymphoma | Brentuximab | Adcetris |
|  |  | Idiopathic pulmonary fibrosis | Pirfenidone | Esbriet |
|  |  | Mantle cell lymphoma | Ibrutinib | Imbruvica |
|  |  |  | Temsirolimus | Torisel |
|  |  | Medullary thyroid carcinoma | Cabozantinib | Cometriq |
|  |  | Multiple myeloma | Lenalidomide | Revlimid |
|  |  |  | Pomalidomide | Imnovid |
|  |  |  | Thalidomide | Thalidomide |
|  |  | Myelodysplastic syndromes | Azacitidine | Vidaza |
|  |  |  | Lenalidomide | Revlimid |
|  |  | Myelodysplastic/myeloproliferative diseases | Imatinib | Glivec |
|  |  | Osteosarcoma | Mifamurtide | Mepact |
|  |  | Ovarian cancer | Olaparib | Lynparza |
|  |  |  | Trabectedin | Yondelis |
|  |  | Pulmonary arterial hypertension | Ambrisentan | Volibris |
|  |  |  | Bosentan | Tracleer |
|  |  |  | Iloprost | Ventavis |
|  |  |  | Macitentan | Opsumit |
|  |  |  | Riociguat | Adempas |
|  |  |  | Sildenafil | Revatio |
|  |  |  | Sitaxentan sodium | Thelin |
|  |  | Renal cell carcinoma | Everolimus | Afinitor |
|  |  |  | Sorafenib | Nexavar |
|  |  |  | Sunitinib | Sutent |
|  |  |  | Temsirolimus | Torisel |
|  |  | Soft tissue sarcoma | Trabectedin | Yondelis |

**Figure S1. Number of European Public Assessment Reports in each cluster**

**Table S3. Dictionary of some methodological terms**

| **Term** | **Meaning** |
| --- | --- |
| Sample size | The total number of subjects studied in a given experiment. |
| Type I error rate | Is the probability of a trial to conclude a difference that actually does not exist in the population from which the sample has been obtained. |
| Type II error rate | Is the probability of a trial to miss a difference that actually exists in the population from which the sample has been obtained. |
| Bias | Bias refers to the tendency to over- or under-estimate the value of a population parameter, such as the treatment effect. It may e.g. be caused by selective sampling or assignment to treatment. |
| Blinding | Is the process to mask the identity of treatments to avoid biases due to knowledge of the treatment identity. When the masking involves both the participating subject and the investigator, it is called double blind. If only the subject is not aware of the the identity of the treatment, it is called single blind. Open label trials are those where all people involved know the identity of the treatment. |
| Randomization | Process of assigning patients to the study arms that includes an element of chance. |
| Stratified randomization | Process of randomization that is executed within subgroups of patients with certain characteristics that may be prognostic for the outcome (such as geographic region or severity of disease). |
| Control group | Group of subjects against who results of the experimental group will be compared. |
| Add on treatment comparison | Design of a trial where the standard of care is received by all subjects in all arms of the study; experimental and control treatments are given on top of the standard of care. |
| Primary endpoint | Main measurement in the trial from which the main conclusions will be derived. |
| Multiplicity of analysis | The number of statistical analysis that are done for a given experiment increase the probability to conclude a positive result, because the overall type 1 error rate is increased with increasing number of analyses. To avoid false conclusions when multiple analysis are foreseen, pre-determined strategies aimed to control the overall type 1 error rate are generally required. |
